# Long-Term Outcomes in Patients with EGFR Positive Lung Adenocarcinoma and Subgroup Analysis Based on Presence of Liver Metastases

**DOI:** 10.3390/cimb46120801

**Published:** 2024-11-24

**Authors:** Vesna Ćeriman Krstić, Ivan Soldatović, Natalija Samardžić, Milija Gajić, Milica Kontić, Aleksandar Reljić, Milan Savić, Marina Roksandić Milenković, Dragana Jovanović

**Affiliations:** 1 Faculty of Medicine, University of Belgrade, 11000 Belgrade, Serbia; milicakontic@yahoo.com (M.K.); drmilansavic@gmail.com (M.S.); 2 Clinic for Pulmonology, University Clinical Center of Serbia, 11000 Belgrade, Serbia; natalis.dm@gmail.com (N.S.); milijagajic@gmail.com (M.G.); 3 Institute of Medical Statistics, Faculty of Medicine, University of Belgrade, 11000 Belgrade, Serbia; soldatovic.ivan@gmail.com; 4 Clinic for Ortopedics, University Clinical Center of Serbia, 11000 Belgrade, Serbia; reljic1993@gmail.com; 5 Clinic for Thoracic Surgery, University Clinical Center of Serbia, 11000 Belgrade, Serbia; 6 Municipal Institute for Lung Diseases and TB, 11000 Belgrade, Serbia; dr.marr@gmail.com; 7 Internal Medicine Clinic “Akta Medica”, 11000 Belgrade, Serbia; draganajv@yahoo.com

**Keywords:** NSCLC, EGFR, liver metastases, immunotherapy, combination therapies, radiotherapy

## Abstract

Lung cancer represents the most common cause of cancer related death. Patients with non-small cell lung cancer (NSCLC) and liver metastases (LM) have worse prognosis with an overall survival (OS) of three to six months. The aim of this study was to investigate long-term outcomes in patients with EGFR mutated (EGFRmut) lung adenocarcinoma as well as the presence of LM. (A total of 105 patients were included in the analysis). They were divided into two groups based on the presence of LM. OS was 13 months for the whole group and also 13 months for patients with and without LM. The 9-year survival rate for patients with and without LM was 12.5% and 3.4%, respectively. Further, the 9-year survival rate for the whole group of patients was 4.8%. There are few data about survival rates beyond 5 years for patients with locally advanced and metastatic EGFRmut NSCLC, mainly because patients with lung cancer rarely live for such a long time. Regarding patients with liver metastases, the results of our study showed similar outcomes compared to patients without LM. As these patients represent a significant number of patients, we need a wider range of therapeutic options. It might be that combination therapies represent a better therapeutic option.

## 1. Introduction

Lung cancer is the most common cause of death among all cancers [1]. Non-small cell lung cancer (NSCLC) represents around 85% of all lung cancers [1]. In the majority of patients, the disease is diagnosed at the locally advanced or metastatic stage [1]. Standard treatment for these patients, in the past, was platinum doublets, with a 5-year survival rate less than 10% [1]. The introduction of molecular therapy in the treatment of NSCLC has led to better clinical outcomes [2,3,4]. It has also been shown that female sex, never having smoked, Asian ethnicity and adenocarcinoma histology could be predictors of a better response to epidermal growth factor receptor (EGFR) tyrosine kinase inhibitor (TKIs) therapy [2,3,4]. More specifically, the type of driver mutation has been shown to have a greater impact on clinical outcomes. Accordingly, it was shown that the best outcomes were found in patients with common mutations, *exon 19 deletion* and *exon 21 L858R* mutation [5,6]. Further, some studies have suggested that patients with *exon 19 deletion* have better outcomes than patients with *exon 21 L858R* mutation [7,8,9] and that patients with the other EGFR mutations—the rare EGFR mutations—have worse outcomes [6]. Recent data showed that patients with *insertion in exon 20* were resistant to EGFR TKIs, with the exception of insertions at amino acid A763 [10,11,12]. However, regardless of the initial response to first- and second-generation EGFR TKIs, it has been shown that acquired resistance has usually developed after 9–13 months [13,14,15]. Additionally, the most common mechanism of resistance, in approximately 60% of cases, is the development of secondary mutation *T790M in exon 20* [16,17].

Although the majority of patients with EGFR mutated (EGFRmut) NSCLC do have a response to EGFR TKIs, 20–30% of patients do not respond to EGFR TKIs or respond for a short time, usually less than 3 months, due to primary resistance to EGFR TKIs [13,15]. It has been suggested that the presence of other co-mutations is the main reason for primary resistance to EGFR TKIs [18,19,20].

Further, patients with EGFRmut NSCLC have poor response to mono-immunotherapy [21,22]. Some studies suggest that the upregulation of the programmed cell death protein 1 (PD-1)/programmed cell death-ligand 1 (PD-L1) pathway is associated with resistance to EGFR TKIs [23,24,25,26]. It has also been reported that high levels of membrane PD-L1 are correlated with a primary resistance and poor response to EGFR TKIs [23,24,25,26]. The results of one study suggested that PD-L1 expression was increased in patients who acquired resistance to EGFR TKIs [24], whereas several other studies have reported a correlation between high PD-L1 expression and primary resistance and poor response to EGFR TKIs [25,26].

Patients with NSCLC and liver metastases (LM) have worse prognosis compared to patients without LM, and they account for approximately one fifth of patients [27]. Studies have shown that these patients live approximately three to six months [27]. The results of one large study which involved more than 20,000 lung cancer patients showed that patients with liver metastases had a 53% higher risk of death compared to patients with brain metastases [28,29]. The majority of them have poor response to chemotherapy [27]. It has also been shown that patients with LM and driver mutations have a worse response to targeted therapies compared to patients without liver metastases [29,30,31], but response to immunotherapy is controversial.

The liver is considered to be an immune-tolerant organ, characterized by T cell anergy and immunosuppressive signals [32]. This could be the reason why patients with NSCLC and liver metastases have a worse response to PD-1/PD-L1 inhibitors compared to patients without LM [27].

## 2. Materials and Methods

### 2.1. Patients and Data Collection

In our study, we enrolled 105 patients with locally advanced and metastatic EGFRmut lung adenocarcinoma, who had been diagnosed at the Clinic for Pulmonology, University Clinical Center of Serbia, from February 2012 until January 2017. All of them were treated with first-line EGFR TKIs-gefitinib, erlotinib or afatinib. All patients met the inclusion criteria—histopathologically confirmed lung adenocarcinoma with confirmed EGFR mutations, locally advanced or metastatic stage of disease, measurable disease at baseline, ECOG PS 0 or 1. The exclusion criteria were as follows: histopathologically confirmed other subtypes of lung carcinoma, early stage of disease, prior treatment for lung adenocarcinoma, ECOG PS ≥ 2. All patients underwent a computed tomography of chest and head or magnetic resonance of the head at baseline to determine the stage of disease. The data about sex, age, smoking status [i.e., non-smokers, ex-smokers (patients who stopped smoking one year before treatment), smokers], stage of disease, response to therapy (ORR), time to disease progression (PFS) and overall survival (OS) were collected. Response to therapy included complete response (CR), partial response (PR), stable disease (SD) and progression of disease (PD). Patients were followed for a minimum of one year (the majority of them until death), and some of them for more than 10 years.

A responder is defined as a patient who had a complete response or partial response for at least 4 weeks (confirmed response). Disease control is defined as response—as defined above—or stable disease for at least 6 weeks. PFS is defined as time from the start of therapy until disease progression or death, whichever occurs first. OS is defined as time from the start of therapy until death.

### 2.2. Statistical Analysis

Results are presented as count (%) or means ± standard deviation, depending on data type. Groups were compared using parametric, *t* test and nonparametric Pearson Chi-square test. The Kaplan–Meier with Log-ranks test was used to assess survival and group differences regarding survival. Survival is presented using the median (95% CI) or percentage of participants without an event of interest in a specific time period. All *p* values less than 0.05 have been considered significant. All data were analyzed using SPSS 29.0 (IBM Corp. Released 2023. IBM SPSS Statistics for Windows, Version 20.0. Armonk, NY, USA: IBM Corp.) and R 3.4.2. (R Core Team (2017). R: A language and environment for statistical computing. R Foundation for Statistical Computing, Vienna, Austria. URL https://www.R-project.org/).

## 3. Results

One hundred and five patients with locally advanced and metastatic EGFRmut lung adenocarcinoma were included in the analysis. Patients were divided into two groups based on the presence of liver metastases. There were 16 patients with LM (15.2%) and 89 patients without LM (84.8%). There were no significant differences between groups by age, gender, smoking status or type of mutation.

The main baseline demographic characteristics for the whole group and for groups based on the presence of liver metastases are presented in Table 1.

Patients with LM achieved disease control in 68.7% of cases, and patients without LM in 77.5% of cases. Fifty percent of patients were responders in the group of patients with LM, and 41.6% in the group of patients without LM. There were no statistically significant differences between groups regarding response to EGFR TKIs (Chi-square test, *p* = 0.206) (Table 2).

**Figure 1:**
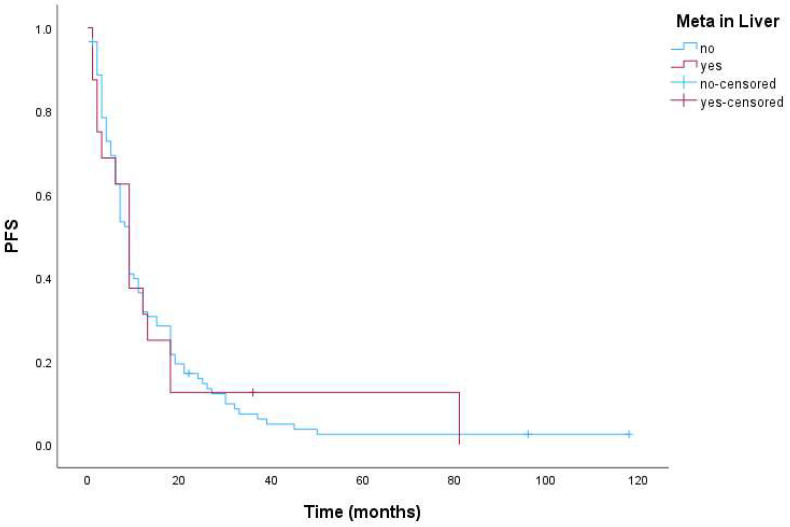
Kaplan–Meier curves for PFS in patients with and without LM (*p* = 0.927).

Progression-free survival was 9 months for the whole group of patients, 95% CI (7.7–10.3). Further, PFS for patients with LM was 9 months, 95% CI (6.2–11.8), and also 9 months, 95% CI (7.6–10.4) for patients without LM. There was no statistically significant difference in PFS between these groups (*p* = 0.927). Figure 1 shows the Kaplan–Meier curves for PFS in patients with and without LM.

**Figure 2:**
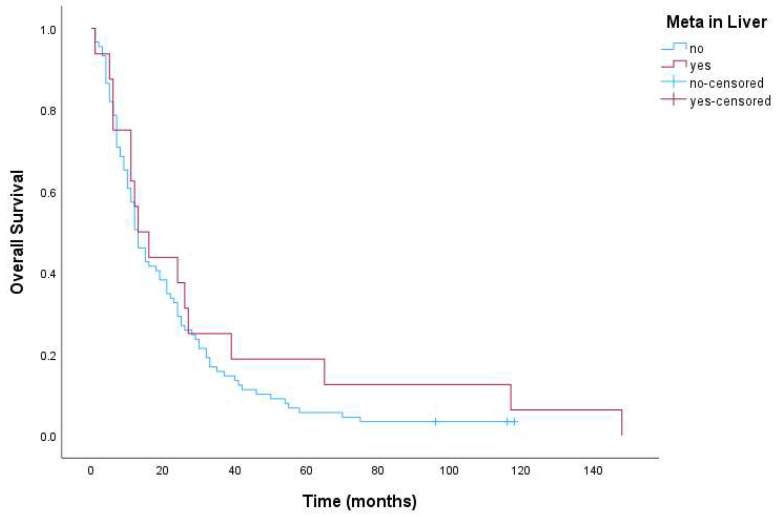
Kaplan–Meier curves for OS in patients with and without liver metastases (*p* = 0.337).

Overall survival was 13 months for the whole group of patients, 95% CI (10.3–15.7). Further, OS for patients with LM was 13 months, 95% CI (10.2–15.8) and also 13 months, 95% CI (5.2–20.8) in the group without LM. There was no statistically significant difference in OS between these groups (*p* = 0.337). Figure 2 shows Kaplan–Meier curves for OS in patients with and without LM.

The 5-year survival rate for patients with and without LM was 12.5% and 4.5%, respectively. The 9-year survival rate for patients with and without LM was 12.5% and 3.4%, respectively. Further, the 5-year and 9-year survival rates for whole group of patients were 6.7% and 4.8%, respectively. The data on 5-year and 9-year OS rates for the whole group, for patients with and without LM and by gender are presented in Table 3.

## 4. Discussion

To the best of our knowledge, this is the second study to have reported this long survival rate for patients with EGFRmut NSCLC treated with first- and second-generation EGFR TKIs, and none of the previous studies reported long-term survival rates for patients with and without LM.

Hirsch et al. [33] reported 10-year and 15-year survival rates of 86% and 59%, respectively, in patients treated with gefitinib, but EGFR mutational status was known for only 22% of patients.

We also found a couple of case reports which reported long-term survival with erlotinib or gefitinib [34,35,36,37,38,39], but only one patient reported by Jovanovic et al. [40] had LM.

Lin et al. [40] reported a 5-year survival rate of 14.6% in patients with EGFRmut NSCLC treated with erlotinib and gefitinib. Another real-world study reported a 5-year survival rate of 21.5% [41].

Tompkins et al. [42] investigated whether patients who were treated with first- and second-generation EGFR TKIs and who lived for 5 years or longer had different demographics and co-mutations compared to patients who lived less than 5 years. The number of co-mutations and the frequency of at least one pathogenic co-mutation did not differ between groups [42]. The most frequent co-mutations were *TP53* and *PIK3CA*, which were found at a similar frequency in both groups [42]. Further, they found that patients with baseline brain metastases had higher odds of death within 5 years, as well as a non-significant trend toward worse OS [42]. Additionally, for patients with baseline liver metastases they found an association with worse 5-year OS but no higher odds of death within 5 years [42]. Patients with EGFRmut NSCLC but not *exon 19 deletion* or *exon 21 L858R* mutation had worse OS and higher odds of death within 5 years [42]. Patients with smoking history also had higher odds of death within 5 years, but smoking history was not associated with worse OS [42].

Several studies investigated the impact of *TP53* mutation on survival in patients with EGFRmut NSCLC. All those studies found that *TP53* was a negative prognostic factor in patients with EGFRmut NSCLC [43,44,45,46].

Jiang et al. [47] investigated whether the addition of local therapy to EGFR-TKIs could provide better outcomes for EGFRmut patients with oligometastatic or oligoprogressive liver metastases compared to EGFR-TKIs alone. The results showed significantly better PFS (13.8 vs. 8.6 months) and OS (31.2 vs. 18.5 months) in the whole group [47]. In the oligometastatic group, there were 23 patients who were treated with EGFR-TKIs and local therapy and 20 patients treated with EGFR-TKIs alone. Patients treated with a combination of EGFR-TKIs and local therapy had significantly better PFS (12.9 vs. 7.9 months) and OS (36.8 vs. 21.3 months) [47].

In another study by Jiang et al. [30], it was investigated whether liver metastasis could be a predictor of EGFR TKI efficacy. There were 598 patients with NSCLC in total, and 99 had liver metastases [30]. Two hundred and ninety-six of them were EGFRmut, and only 56 of EGFRmut received EGFR TKIs in a first-line setting. In the EGFRmut group, patients who had LM and who were treated with EGFR TKIs had shorter PFS (7.5 vs. 11.8 months) and OS (20.8 vs. 30.6 months) compared to patients without liver metastases [30]. Patients with liver metastases who were treated with first-line chemotherapy, regardless of EGFR status, had similar PFS and OS compared to patients without liver metastases [30].

Similar results were obtained in a study conducted by Wu et al. [31]. There were 148 patients included in the analysis; among them, 19 patients had LM (13%) [31]. Patients with liver metastases who had been treated with gefitinib had shorter PFS and OS compared to those without liver metastases [31]. It was shown by multivariable analysis that liver metastases were an independent poor prognostic factor for PFS and OS [31].

Chang et al. [48] conducted a study the results of which showed similar OS in patients with EGFRmut and EGFRwt NSCLC who had baseline liver metastases. Patients with EGFRmut NSCLC were all treated with TKIs in first line, and patients with EGFRwt NSCLC were treated with chemotherapy or best supportive care if they had poor performance status [48]. A total of 490 patients were included, and among them 39 had EGFRmut NSCLC and LM [48]. Liver metastases were a poor prognostic factor in patients with EGFRmut NSCLC, but it did not have an impact on OS in patients with EGFRwt NSCLC [48].

In another large study that included more than 2000 patients with EGFRmut NSCLC, 13.2% of patients had baseline liver metastases [49]. It was also shown that these patients had a lower response to treatment with first- and second-generation EGFR TKIs [49].

A retrospective analysis evaluated the clinical outcomes in 236 patients with metastatic NSCLC [50]. Patients with liver metastases accounted for 16.9% of all patients, and they had lower OS compared to patients without liver metastases (10 vs. 21 months, respectively) [50]. In a subgroup analysis, patients with EGFRmut NSCLC and liver metastases had better OS compared to patients with EGFRwt NSCLC (not reached vs. 13 months) [50].

A RELAY study showed a better HR of PFS with the addition of ramucirumab (anti-VEGF receptor antibody) to erlotinib in patients with liver metastases compared to erlotinib alone [51].

Further, Chiu et al. [52] investigated which factors were associated with prolonged survival (>36 months) in EGFRmut advanced NSCLC patients treated with first-line afatinib. They found that liver metastases were independently and negatively associated with prolonged PFS [52].

Gen et al. [53] investigated the efficacy of osimertinib in EGFRmut metastatic NSCLC compared to first-/second-generation EGFR TKIs. Clinical data for 388 patients were retrospectively evaluated, and 8.8% of them had liver metastases [53]. In the osimertinib group, patients with liver metastases had significantly lower PFS and OS compared to patients without (7.4 vs. 19.7 months, and 12.1 vs. not reached) [53]. In the gefitinib/erlotinib group and afatinib group, the presence of liver metastases was not associated with shorter PFS [53]. In addition, PFS in patients with liver metastases was not significantly different among three groups [53]. The ORR of osimertinib was significantly lower in patients with liver metastases compared to those without, and it was similar to the ORR of gefitinib, erlotinib and afatinib [53].

Similar results for osimertinib efficacy in patients with liver metastases were shown in a retrospective study by Sakata et al. [54].

Another study investigated the efficacy of osimertinib according to mutation subtypes and different sites of baseline metastases [55]. Fourteen percent of patients had liver metastases, and it was shown that those patients had shorter PFS compared to patients without liver metastases (10.3 vs. 24.1 months, respectively) [55].

A comparison of clinical outcomes for patients treated with EGFR TKIs in some studies is presented in Table 4.

The results of the Mariposa study showed significantly better PFS for patients with advanced EGFRmut lung adenocarcinoma who were treated with amivantanab-lazertinib compared to osimertinib in a first-line setting [56]. Subgroup analysis also showed that patients with liver metastases had better PFS if they were treated with amivantanab-lazertinib compared to osimertinib (18.2 vs. 11 months) [56].

An ImPower150 study [57] investigated the efficacy of different therapeutic options in patients with metastatic NSCLC in first-line treatment. Patients with EGFRmut (*deletion 19* or *exon 21 L858R*) NSCLC and patients with ALK translocations were also included, but it was required that patients have disease progression or treatment intolerance with at least one TKI [57]. It was shown that patients with liver metastases had better outcomes if they were treated with bevacizumab (VEGF inhibitor), atezolizumab (PD-L1 inhibitor) and chemotherapy compared to those who were treated with bevacizumab and chemotherapy alone (PFS 7.4 vs. 4.9 months; OS 13.3 vs. 9.4 months) [58]. There was no difference in outcomes for patients who were treated with atezolizumab and chemotherapy compared to those treated with bevacizumab and chemotherapy [58]. In a group of patients with sensitizing EGFR mutations, OS was longer in a group of patients who were treated with bevacizumab, atezolizumab and chemotherapy compared to patients who were treated with bevacizumab and chemotherapy (OS 29.4 vs. 18.1 months, HR 0.60) [57]. Further, OS was similar in patients who were treated with atezolizumab and chemotherapy compared to those treated with bevacizumab and chemotherapy (OS 19.0 vs. 18.1 months, HR 1.00) [57]. The 3-year survival rate was 41.9% for patients treated with bevacizumab, atezolizumab and chemotherapy, 25.6% for patients treated with atezolizumab and chemotherapy and 24.6% for patients treated with bevacizumab and chemotherapy [57].

One retrospective study evaluated the efficacy of immunotherapy + chemotherapy, immunotherapy + chemotherapy + antiangiogenic therapy and chemotherapy + antiangiogenic therapy in EGFRmut patients after the failure of EGFR TKIs [58]. Liver metastases were found in 29% of patients [58]. The results showed the superior PFS of immunotherapy + chemotherapy + antiangiogenic therapy in this subgroup of patients compared to other treatment options (6.44 months vs. 0.95 months for patients treated with immunotherapy + chemotherapy and 3.48 months for patients treated with chemotherapy + antiangiogenic therapy) [58].

As was mentioned previously, the liver is considered to be an immune-tolerant organ, and it is likely that liver metastases will be resistant to immunotherapy [32]. Some studies suggested that liver metastases could actively induce the apoptosis of CD8+ T cells after their interaction with macrophages, which could result in liver metastases taking over mechanisms of peripheral tolerance and lead to an acquired resistance to immunotherapy through the removal of CD8+ T cells [32].

PD-L1 is an immune checkpoint that limits T cell activity by binding to its receptor PD-1 on activated T cells [32]. PD-L1 can also be found on tumor cells and that way blocks the activity of the immune system to fight against various cancers [32]. Furthermore, blocking PD-1/PD-L1 leads to a reactivation of exhausted T cells and to a reactivation of the immune system to fight against tumors [32]. The tumor as well as the organ specific microenvironment plays an important role in this process [32]. Vascular endothelial growth factor (VEGF) could block antitumor activity by promoting vascularization [59], which is often used by tumors for growth and metastasis, and interrupting T cell infiltration into the tumor [60,61]. It has been suggested that VEGF inhibitor in combination with chemotherapy could enhance PD-L1 inhibitor-induced T cell-mediated cancer cell death, by reversing VEGF-induced immunosuppression by promoting the infiltration of T cells into the tumor microenvironment and enabling T cell activity against tumor antigens, as well as chemotherapy-induced cancer cell death [57].

It could be assumed that susceptibility to VEGF inhibitor in patients with NSCLC and liver metastases arises from a reversal of immune suppression by VEGF inhibitor, which basically reduces the infiltration of CD8+ T cells, which may further enhance the T cell-mediated killing of cancer cells by PD-L1 inhibitor [57]. The efficacy of this has been shown regardless of PD-L1 expression [57]. It could be explained by the fact that activating both mechanisms—T cell activation by atezolizumab and the interferon γ-mediated induction of PD-L1 expression—throw the reprogramming tumor microenvironment into the active immune system by VEGFR inhibitor [57]. The efficacy of bevacizumab and atezolizumab in combination with chemotherapy in patients with NSCLC and liver metastases is supported by an investigation in patients with unresectable hepatocellular carcinoma who were treated with bevacizumab and atezolizumab in combination with chemotherapy [57]. It seems that patients with NSCLC and liver metastases respond to treatment similarly to patients with primary liver cancer [57].

In the past few years, there have been a large number of papers investigating the role of tumor-educated platelets as a biomarker for the early diagnosis of tumors, including NSCLC, as well as their potential role in the early detection of tumor progression. Platelet transcriptome is a product of parental megakaryocyte profile and local and systemic pathological conditions like cancer, which can modulate their transcriptome [62,63]. Thus, tumor cells can educate platelets and “leave their signature”, which can lead to diagnosis [62,63]. In the interaction process with tumor cells, platelets can modify their RNA profile and thus derive tumor-educated platelets which produce a different microenvironment from the normal one [62,63].

Several studies were conducted which included different platelet RNA in patients with NSCLC. It was shown that tumor-educated platelet RNA may enable an early diagnosis of NSCLC and an early detection of progression and may serve as a potential biomarker of response to chemotherapy [62].

However, EGFR mutations could not be detected in the DNA of tumor-educated platelets isolated from patients with NSCLC [64].

## 5. Conclusions

There are few data about survival rates beyond 5 years for patients with locally advanced and metastatic EGFRmut NSCLC, mainly because patients with lung cancer rarely live for a very long time. Regarding patients with liver metastases, the results of other studies have shown that they have worse prognosis compared to other groups of patients regardless of the applied treatment. In our study, clinical outcomes were similar in patients with EGFRmut NSCLC with and without LM, but, in our study, there were a small number of patients with LM. Moreover, patients were treated with first-/second-generation TKIs, but in some countries these drugs are still first-line treatment options. As these patients represent a significant number of patients, we need a wider range of therapeutic options. It might be that combination therapies represent better a therapeutic option for this subset of patients.

## Figures and Tables

**Table 1:** Comparative view of demographic characteristics for the whole group and for patients with and without liver metastases.

Demographic Characteristics	All Patients *N* = 105	Patients with Liver Metastases *N* = 16	Patients Without Liver Metastases *N* = 89	* p * Value
Age, mean ± sd	63.7 ± 10.6	62.4 ± 8.7	63.9 ± 10.9	*p* = 0.603 ^a^
Gender				*p* = 1.000 ^b^
Female sex, ***n*** (%)	74 (70.5%)	11 (69%)	63 (71%)	
Male sex, ***n*** (%)	31 (29.5%)	5 (31%)	26 (29%)	
Smoking, ***n*** (%)				*p* = 0.487 ^b^
Never smokers	60 (57.7%)	11 (69%)	49 (55%)	
Former smokers	17 (16.3%)	1 (6%)	16 (18%)	
Current smokers	27 (26.0%)	4 (25%)	23 (26%)	
EGFR mutation				*p* = 0.248 ^b^
***Del 19***	54 (51.4%)	7 (44%)	47 (53%)	
***L858R point mutation exon 21***	35 (33.3%)	8 (50%)	27 (30%)	
Rare mutations	16 (15.2%)	1 (6%)	15 (17%)	
***Del 19*** and ***T790M***	1	1		
***Del 20***	1		1	
***Exon 18***	1		1	
***G719X exon 18***	3		3	
***G719X exon 18*** and ***S768I exon 20***	2		2	
***Exon 20 insertion***	7		7	
***L828V***	1		1	

^a^ Independent samples *t* test; ^b^ Pearson chi square test.

**Table 2:** Response to EGFR TKIs by group (*p* = 0.206).

Variable	Patients with LM N = 16	Patients Without LM N = 89
Complete response	1/16 (6.3%)	2/89 (2.2%)
Partial response	7/16 (43.8%)	35/89 (39.3%)
**Response**	**8/16 (50%)**	**37/89 (41.6%)**
Stable disease	3/16 (18.8%)	32/89 (36.0%)
**Disease control**	**11/16 (68.7%)**	**69/89 (77.5%)**
**Disease progression**	**5/16 (31.3%)**	**20/89 (22.5%)**

**Table 3:** Comparative view of 5-year and 9-year OS rate.

Patients	5-Year OS Rate	9-Year OS Rate	Female No (%)	Female 5-Year OS Rate	Female 9-Year OS Rate	Male No (%)	Male 5-Year OS Rate	Male 9-Year OS Rate
**Whole group of patients**	6.7%	4.8%	74 (70.5%)	6.8%	4%	31 (29.5%)	9.7%	6.5%
**Patients with LM**	12.5%	12.5%	11 (31%)	18.2%	18.2%	5 (69%)	20%	0%
**Patients without LM**	4.5%	3.4%	63 (70.2%)	4.8%	1.6%	26 (29.2%)	7.7%	7.7%

**Table 4:** Comparison of clinical outcomes for patients treated with EGFR TKIs in some studies.

Study	No of Patients	OS (Months)	PFS (Months)	5-Year OS Rate	9-Year OS Rate
**Hirsh et al.** [33] *	79	/	/	/	10-year OS rate 86%, 15-year OS rate 59%
**Lin et al.** [40]	137	30.9	12.1	14.6%	/
**Chiu et al.** [52]	546	27.2	14.5	/	/
**Gen et al.** [53]	388				
**Sakata et al.** [54]	538	/	20.5	/	/
**Takeyasu et al.** [55]	229	33.7	23.3	/	/
**Ćeriman Krstić et al.**	105	13	9	6.7%	4.8%

* EGFR ststus was known for 17 patients (22%).

## Data Availability

The data presented in this study are available upon reasonable request from the corresponding author (accurately indicating status).
